# Supplementary material for: pH-responsive high stability polymeric nanoparticles for targeted delivery of anticancer therapeutics
Source: Commun Biol. 2020 Mar 3;3:95. doi: 10.1038/s42003-020-0817-4 (PMC7054360; doi:10.1038/s42003-020-0817-4)
Supplement: Supplementary file 1 — Supplementary Information [file 42003_2020_817_MOESM1_ESM.pdf]

Table of Contents

Supporting Figures

Supplementary Figure 1. Synthesis and characterization of doxorubicin-triphenylphosphine (Dox-TPP)
Supplementary Figure 2. ^{1}H NMR spectrum of Dox-TPP
Supplementary Figure 3. Cell viability of MCF-7 cells treated with unmodified Dox and Dox-TPP
Supplementary Figure 4. Synthetic scheme for preparation of BSA-PLGA nanoparticles (NPs)
Supplementary Figure 5. Characterization of the BSA-PLGA NPs
Supplementary Figure 6. Effects of high serum concentration and high ionic strength on colloidal stability of BSA-PLGA NPs
Supplementary Figure 7. Encapsulation stability of the BSA-PLGA NPs in solution
Supplementary Figure 8. Encapsulation stability of the BSA-PLGA NPs in cells
Supplementary Figure 9. UV-Vis spectroscopy evaluation of conjugation of the ATRAM peptide to the surface of the BSA-PLGA NPs
Supplementary Figure 10. Representative binding curve of ATRAM-NBD to BSA measured by fluorescence anisotropy
Supplementary Figure 11. Confocal laser scanning microscopy images of HeLa cells incubated with Dox-TPP loaded ATRAM-BSA-PLGA NPs
Supplementary Figure 12. Gating strategy for flow cytometry analysis
Supplementary Figure 13. Confocal laser scanning microscopy images of MCF-7 cells incubated with Dox-TPP loaded BSA-PLGA NPs
Supplementary Figure 14. Effects of low temperature on uptake of ATRAM-BSA-PLGA NPs
Supplementary Figure 15. Cell viability of MCF-7 cells treated with drug-free ATRAM-BSA-PLGA NPs at pH 6.5
Supplementary Figure 16. Cell viability of MCF-7 and HeLa cells treated with Dox-TPP loaded BSA-PLGA NPs
Supplementary Figure 17. Cell viability of MCF-7 cells treated with paclitaxel (PTX)-loaded ATRAM-BSA-PLGA NPs
Supplementary Figure 18. Confocal laser scanning microscopy images of differentiated human monocytic leukemia THP-1 cells treated with Dox-TPP loaded BSA-PLGA NPs
Supplementary Figure 19. Effects of drug-free ATRAM-BSA-PLGA NPs on tumor volume and body weight of 4T1 tumor-bearing mice
Supplementary Figure 20. Body weight changes of 4T1 tumor-bearing mice following treatment with Dox-TPP loaded ATRAM-BSA-PLGA NPs
Supplementary Figure 21. Histological analysis of vital organs following treatment with Dox-TPP loaded ATRAM-BSA-PLGA NPs

Supporting Tables

Supplementary Table 1. Drug loading capacity of BSA-PLGA NPs
Supplementary Table 2. Diameters and zeta potentials of PLGA, BSA-PLGA (with and without crosslinking) and ATRAM-BSA-PLGA NPs nanoparticles
Supplementary Table 3. Proteins corresponding to the UniProt Knowledgebase (UniProtKB) accession numbers shown in Figure 2

Supporting Notes

Supplementary Note 1. Doxorubicin-triphenylphosphonium (Dox-TPP) and multidrug resistance (MDR)
Supplementary Note 2. Glutathione (GSH)-mediated cargo release from BSA-PLGA NPs
Supplementary Note 3. pK_a of acidity-triggered rational membrane (ATRAM) peptide
Supplementary Note 4. Tumor-associated macrophages

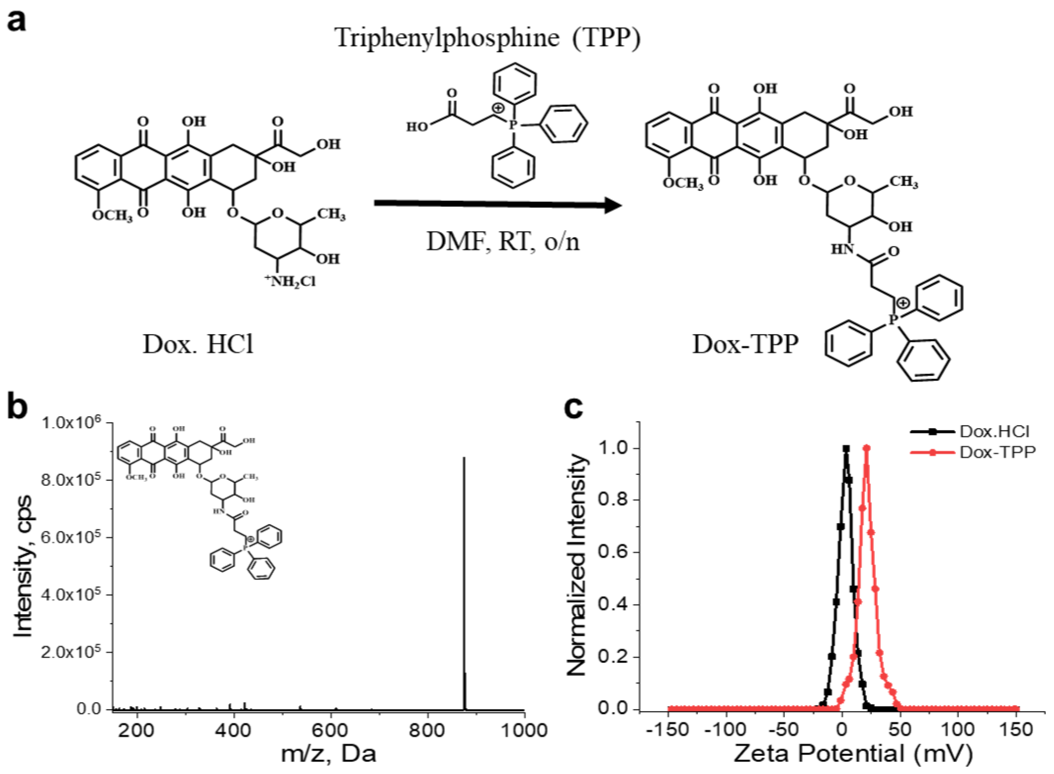

Supplementary Figure 1. Synthesis and characterization of doxorubicin-triphenylphosphine (Dox-TPP). (**a**) Synthetic scheme for preparation of Dox-TPP.[1] (**b**) Characterization of Dox-TPP using LC-MS. Due to the negative charge, the bromine ion did not appear in the spectrum, and the counter ion peak was observed at 874.4 ($C_{49}H_{50}NO_{12}P^+$). The M-1 peak at 873.4 was strong compared to the molecular ion peak, which is likely a result of loss of a hydrogen radical from either the acetal group or one of the benzyl groups in M^+. (**c**) Zeta potential measurements of Dox and Dox-TPP solutions (40 µg mL^{-1}).

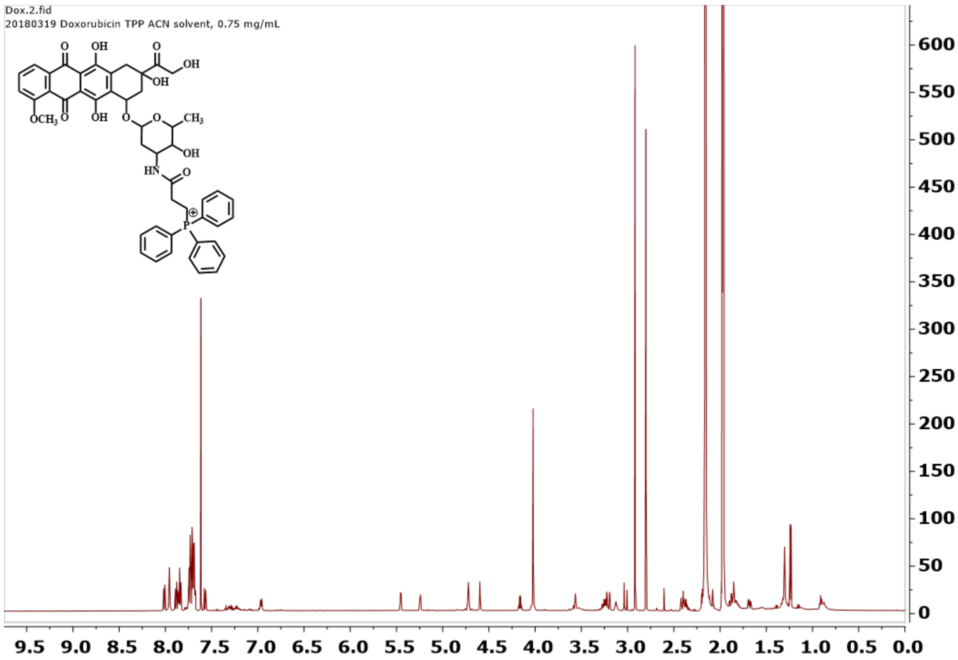

Supplementary Figure 2. ^{1}H NMR spectrum of Dox-TPP. δ: 14.08 (brs, 1H, O-H), 13.31 (brs, 1H, O-H) 8.00 (d, J = 6.4Hz, 1H, Ar-H), 7.95 (brs, 2H, Ar-H), 7.82 – 7.89 (m, 3H, Ar-H), 7.67-7.74 (m, 10H Ar-H), 7.56 (d, J = 6.7 Hz, 1H, Ar-H), 6.95 (d, J = 6.75 Hz, 1H, Ar-H), 5.45 (d, j = 3 Hz 2H), 5.23 (ABq, j = 1.9 Hz, 1H), 4.72 (t, j = 3 Hz, 2H), 4.60 (s, 1H), 4.14 (ABq, j = 5.4 Hz, 2H), 4.02 (s, 3H), 3.56 (s, 3H), 3.19-3.26 (m, 4H),3.00-3.12 (m, 3H), 1.30 (brs, 2H) 1.23 (d, j = 5.5 Hz, 3H).

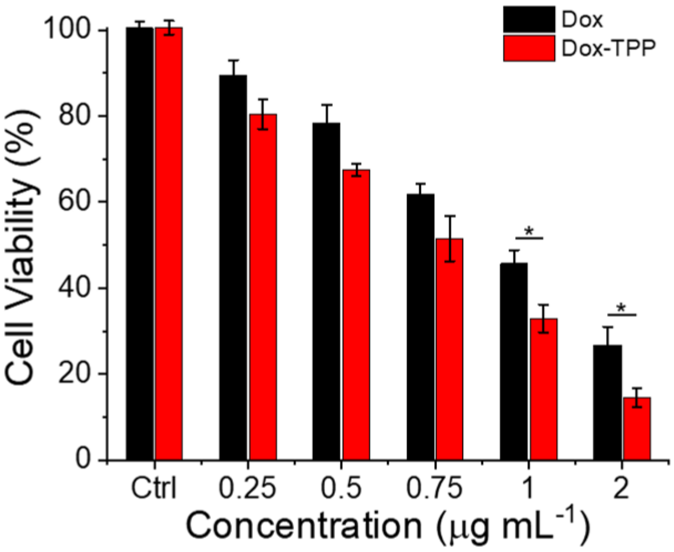

Supplementary Figure 3. Cell viability of MCF-7 cells treated with unmodified Dox and Dox-TPP for 48h. Cell viability was assessed using the MTS assay, with the % viability determined form the ratio of the absorbance of the treated cells to the control cells (n = 4). *$P < 0.05$.

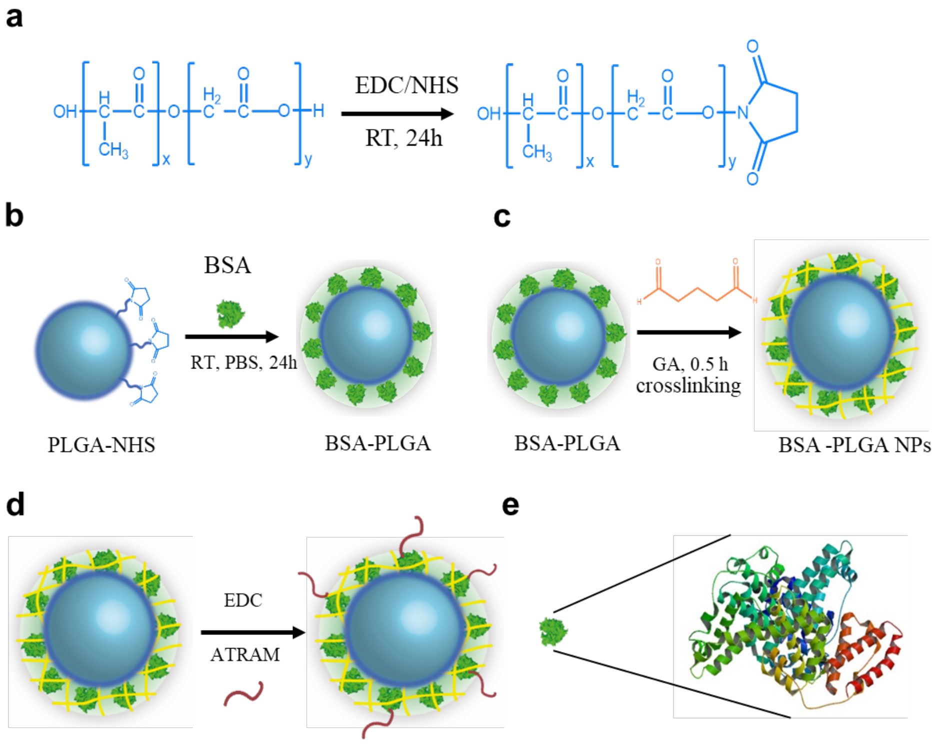

Supplementary Figure 4. Synthetic scheme for preparation of BSA-PLGA nanoparticles (NPs): (**a**) activation of polylactic-co-glycolic acid (PLGA) NPs with carbodiimide (EDC)/N-hydroxysuccinimide (NHS) towards functionalization of the sucinnamide functional groups; (**b**) amine conjugation chemistry to covalently couple bovine serum albumin (BSA) to the surface of PLGA-NHS; (**c**) crosslinking the BSA on the surface of the NPs using glutaraldehyde (GA) to increase the encapsulation stability; (**d**) functionalizing the BSA on the surface with acidity-triggered rational membrane (ATRAM) peptide using simple EDC coupling; (**e**) crystal structure of BSA (source: RCSB PDB 3VO3).

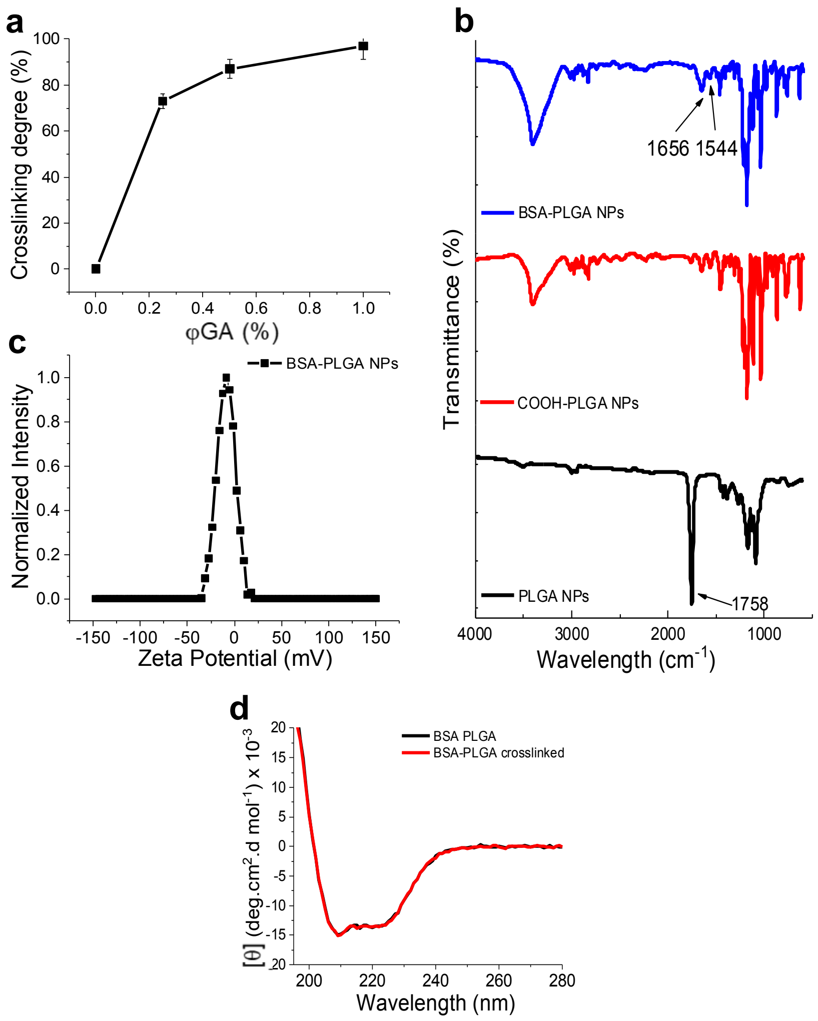

Supplementary Figure 5. Characterization of the BSA-PLGA NPs. (**a**) Extent of BSA crosslinking on the surface of the NPs as a function of glutaraldehyde (GA) concentration (w/w%) was determined using the trinitrobenzenesulfonic acid (TNBS) assay.[2] (**b**) FTIR spectra of PLGA-COOH and PLGA and BSA-PLGA NPs. The FTIR spectrum of the BSA-PLGA sample shows transmittance peaks at 1656 cm^{-1} and 1544 cm^{-1}, which are assigned to the stretching vibration of C=O (amide bond I) of PLGA and bending vibration of N-H (amide bond II) of BSA, respectively.[3] The transmittance peak at 1758 cm^{-1} is assigned to the stretching vibration of C=O ester bond in PLGA, which is utilized for BSA coupling via amide bond formation.[4] Finally, the transmittance peak at 3400 cm^{-1} is assigned to O-H stretching in the BSA-PLGA NPs.[5] (**c**) Zeta potential measurement for BSA-PLGA NPs in cell culture medium containing 10% FBS. (**d**) CD spectra of BSA, BSA-PLGA and crosslinked BSA-PLGA NPs.

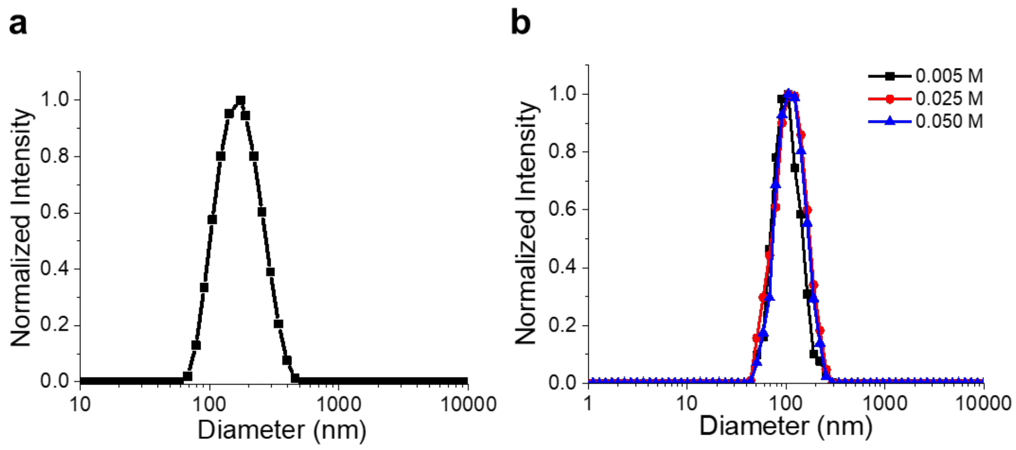

Supplementary Figure 6. Effects of high serum concentration and high ionic strength on colloidal stability of crosslinked BSA-PLGA NPs. Size analysis of BSA-PLGA NPs incubated for 72 h in: (**a**) cell culture medium containing 50% fetal bovine serum (FBS), or (**b**) 1×PBS (137 mM NaCl, 2.7 mM KCl, 8 mM Na_2HPO_4, and 2 mMKH_2PO_4) containing increasing concentrations of NaCl.

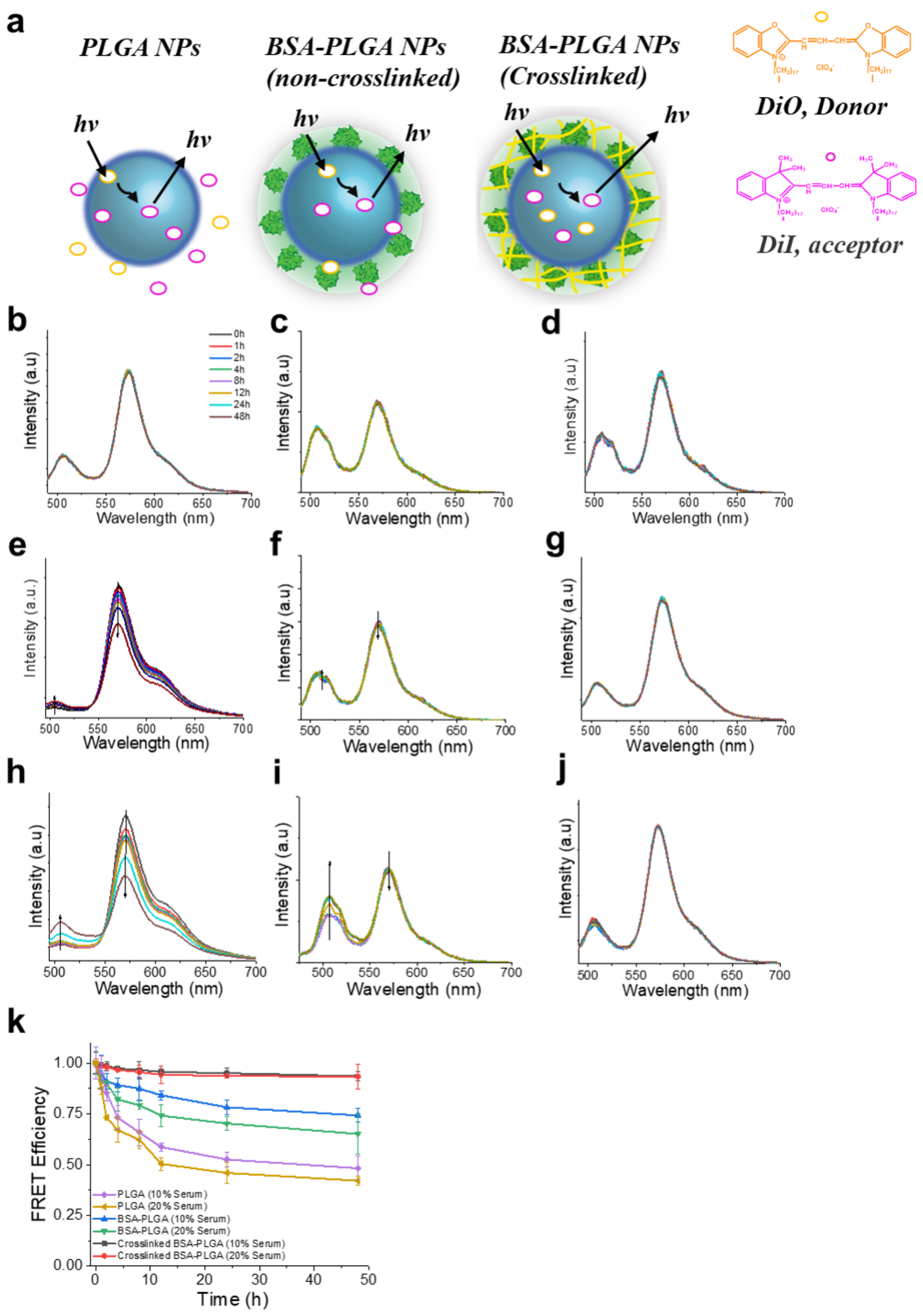

Supplementary Figure 7. Encapsulation stability of the BSA-PLGA NPs in solution. (**a**) Schematic representation of the FRET-based encapsulation stability analysis for PLGA, non-crosslinked BSA-PLGA and crosslinked BSA-PLGA NPs co-loaded with DiO (donor, λ_{em} = 505 nm) and DiI (acceptor, λ_{em} = 565 nm) fluorophores. (**b–j**) FRET efficiency analysis for PLGA (**b**,**e**,**h**), non-crosslinked BSA-PLGA (**c**,**f**,**i**) and crosslinked BSA-PLGA (**d**,**g**,**j**) NPs in 10 mM phosphate buffer solution at pH 7.4 (**b**,**c**,**d**), or cell culture medium containing 10% (**e**,**f**,**g**) or 20% (**h**,**i**,**j**) FBS. The excitation wavelength was set at 450 nm, and the fluorescence spectra were recorded at different time points over 48 h. (**k**) Summary of time-dependence of FRET efficiency (=$I_{DiI}/(I_{DiI} + I_{DiO})$) for PLGA, non-crosslinked BSA-PLGA and crosslinked BSA-PLGA NPs under the conditions described in (**e–j**) (n = 4).

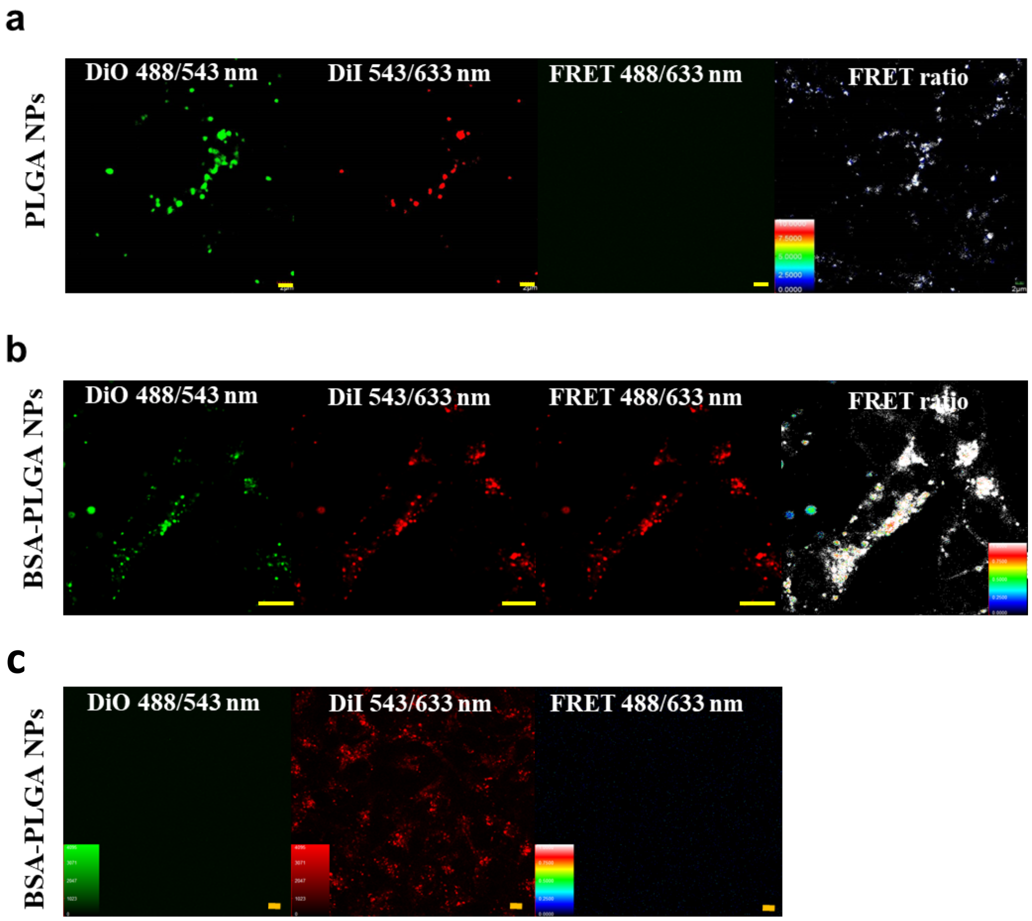

Supplementary Figure 8. Encapsulation stability of the BSA-PLGA NPs in cells. Confocal laser scanning microscopy images of MCF-7 cells incubated with DiO/DiI co-loaded PLGA (**a**) or crosslinked BSA-PLGA (**b**) NPs, or DiI loaded crosslinked BSA-PLGA NPs (**c**), for 4 h at pH 7.4. Confocal microscope fluorescence parameters: DiO, $\lambda_{ex/em}$ = 488/543 nm; DiI, $\lambda_{ex/em}$ = 543/633 nm; FRET, $\lambda_{ex/em}$ = 488/633 nm; FRET ratio = $I_{FRET}/(I_{FRET} + I_{DiO})$. Scale bar = 10 μm.

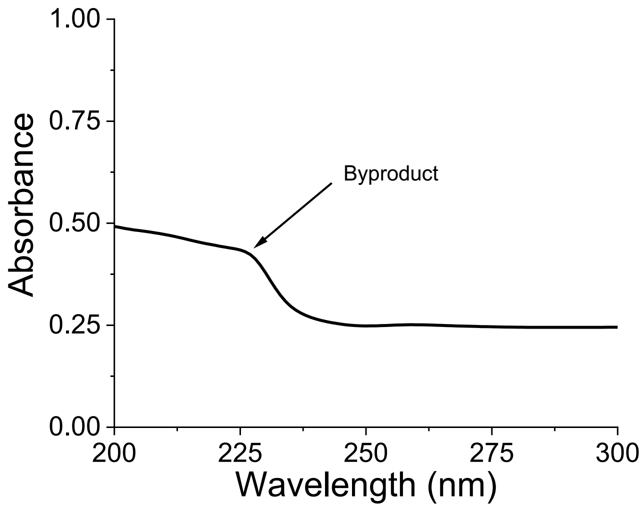

Supplementary Figure 9. UV-Vis spectroscopy evaluation of conjugation of the ATRAM peptide to the surface of the BSA-PLGA NPs by release of the urea byproduct at 232 nm.[6]

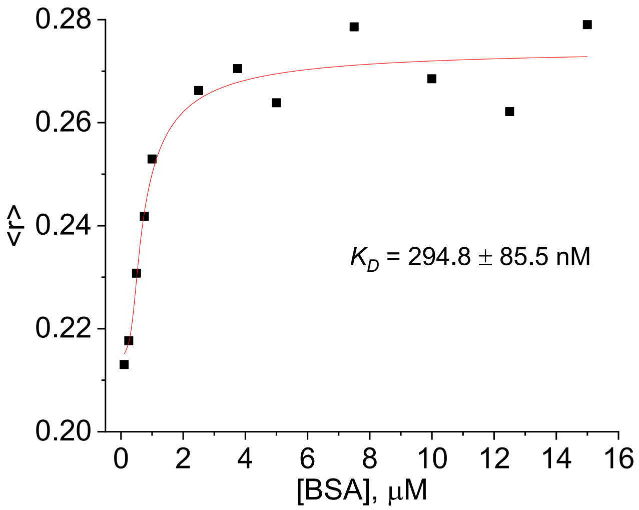

Supplementary Figure 10. Representative binding curve of ATRAM-NBD to BSA measured by fluorescence anisotropy. The curve was fitted to *Equation 3* to determine the dissociation constant.[7]

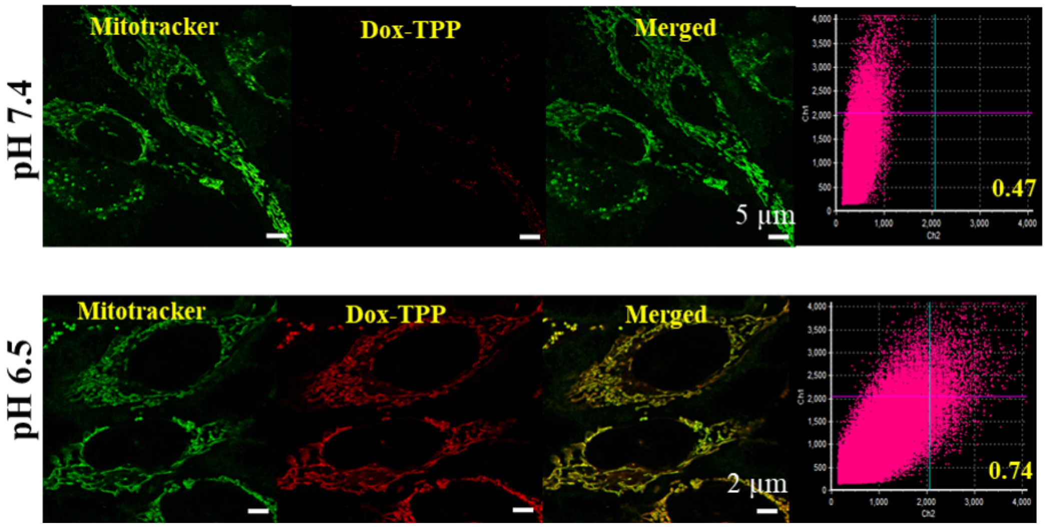

Supplementary Figure 11. Confocal laser scanning microscopy images of HeLa cells incubated with Dox-TPP loaded ATRAM-BSA-PLGA NPs for 1 h at pH 7.4 or 6.5. Inset: analysis of colocalization of the Dox-TPP cargo (red) with mitochondria (MitoTracker Green) using Pearson's correlation coefficient. Scale bar = 5 and 2 µm.

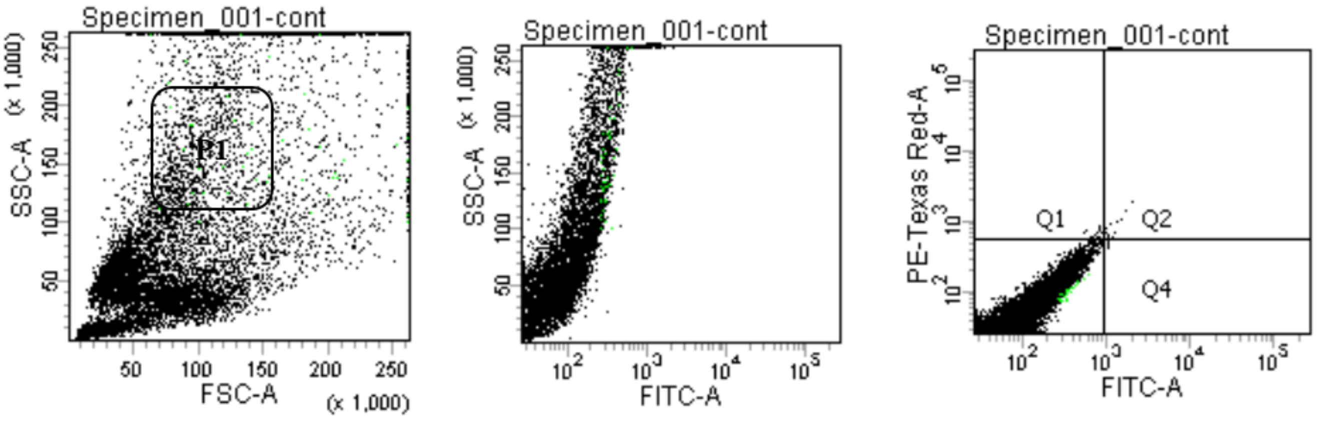

Supplementary Figure 12. Gating strategy for flow cytometry analysis. Typical exclusion gating to eliminate dead cells and debris from the analysis.

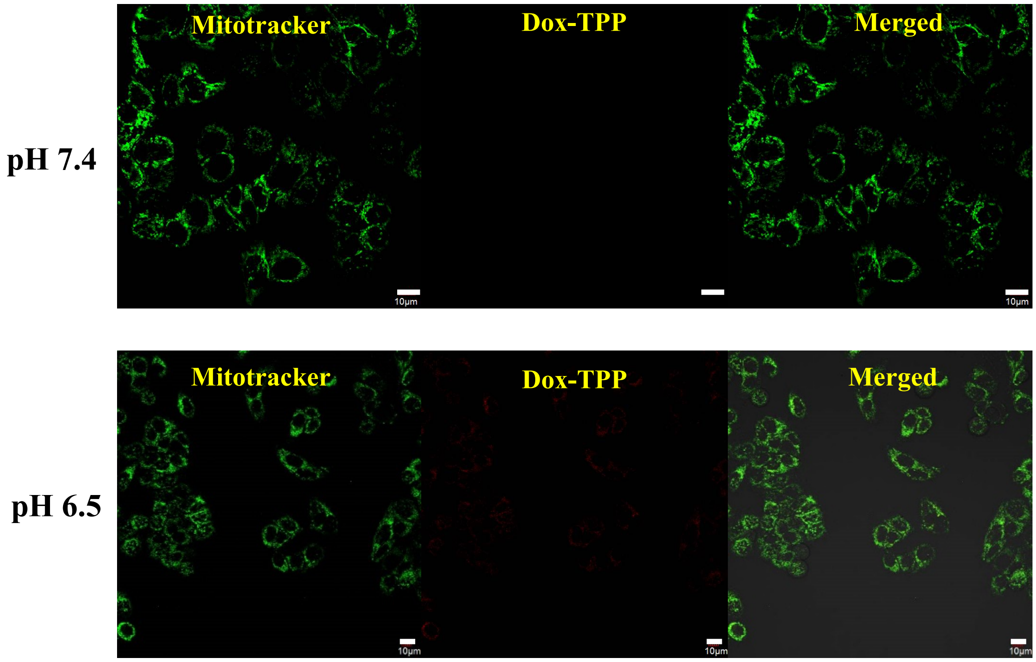

Supplementary Figure 13. Confocal laser scanning microscopy images of MCF-7 cells incubated with Dox-TPP loaded BSA-PLGA NPs for 1 h at pH 7.4 or 6.5. Scale bar = 10 µm.

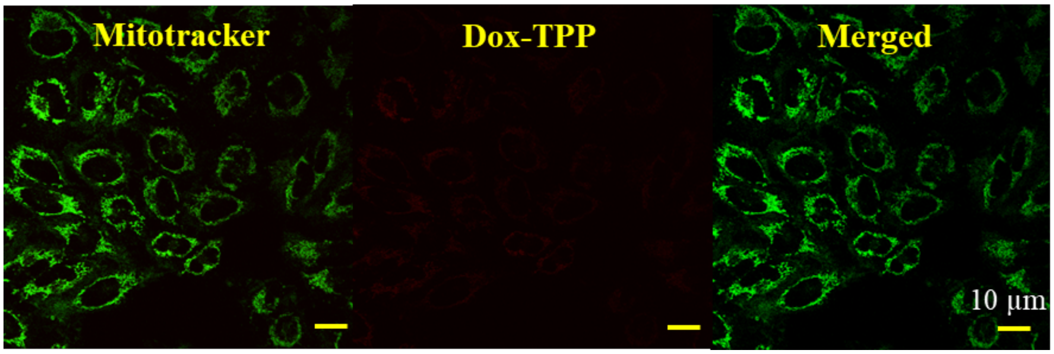

Supplementary Figure 14. Effects of low temperature on uptake of ATRAM-BSA-PLGA NPs. Confocal laser scanning microscopy images of MCF-7 cells incubated with Dox-TPP loaded ATRAM-BSA-PLGA NPs for 1 h at 4 ºC. Scale bar = 10 µm.

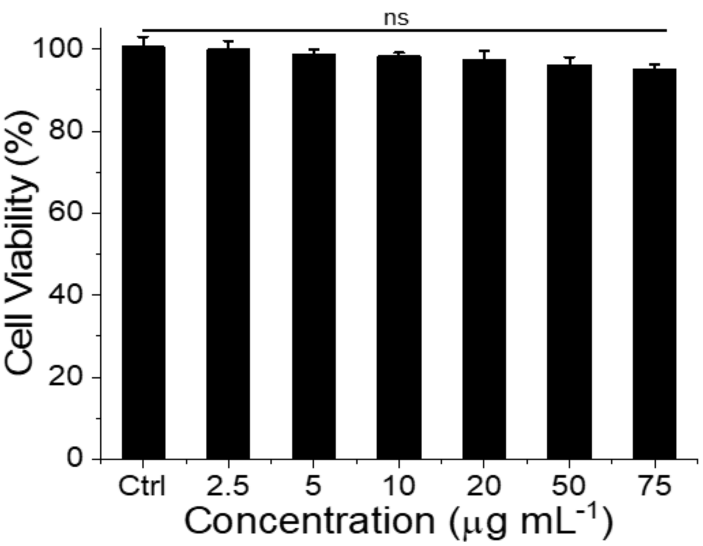

Supplementary Figure 15. Cell viability of MCF-7 cells treated with drug-free ATRAM-BSA-PLGA NPs for 48 h at pH 6.5. Cell viability was assessed using the MTS assay, with the % viability determined form the ratio of the absorbance of the treated cells to the control cells (n = 4). ns, non-significant ($P >$ 0.05).

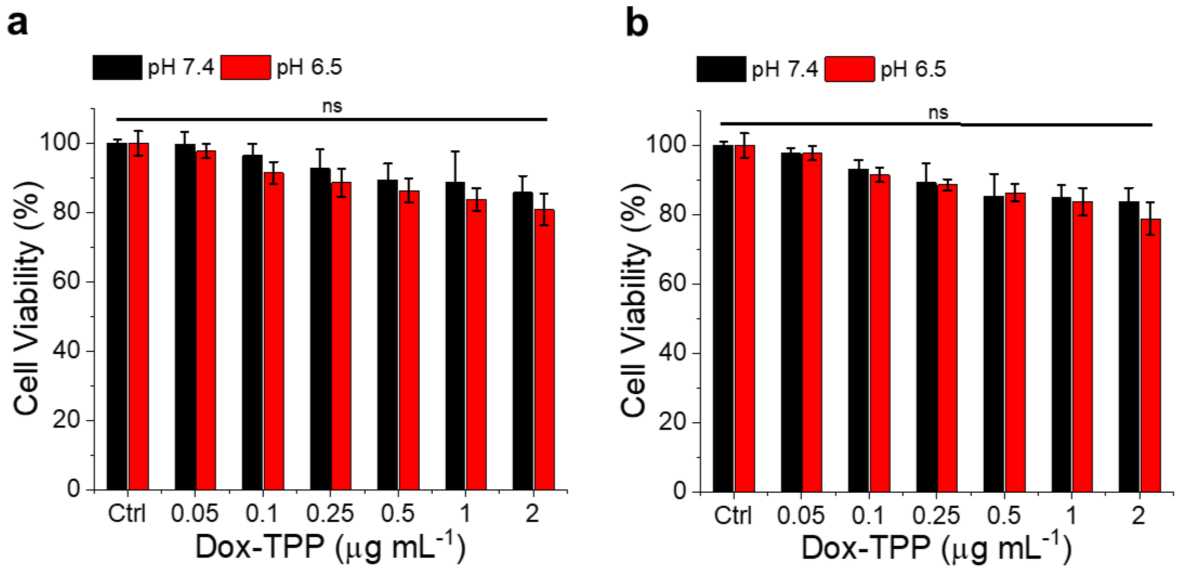

Supplementary Figure 16. Cell viability of MCF-7 (**a**) and HeLa (**b**) cells treated with Dox-TPP loaded BSA-PLGA NPs for 48 h at pH 7.4 or 6.5. Cell viability was assessed using the MTS assay, with the % viability determined form the ratio of the absorbance of the treated cells to the control cells (n = 4). ns, non-significant ($P > 0.05$).

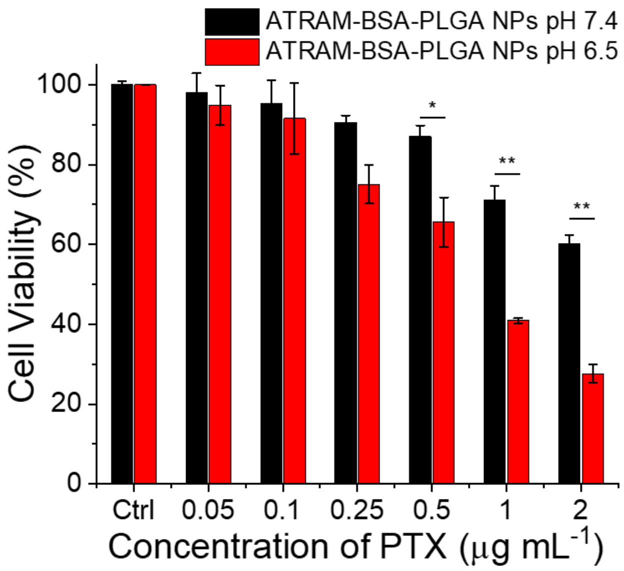

Supplementary Figure 17. Cell viability of MCF-7 cells treated with paclitaxel (PTX)-loaded ATRAM-BSA-PLGA NPs for 48h at pH 7.4 or pH 6.5. Cell viability was assessed using the MTS assay, with the % viability determined form the ratio of the absorbance of the treated cells to the control cells (n = 4). $^*P < 0.05$, $^{**}P < 0.01$.

1h
2h
4h

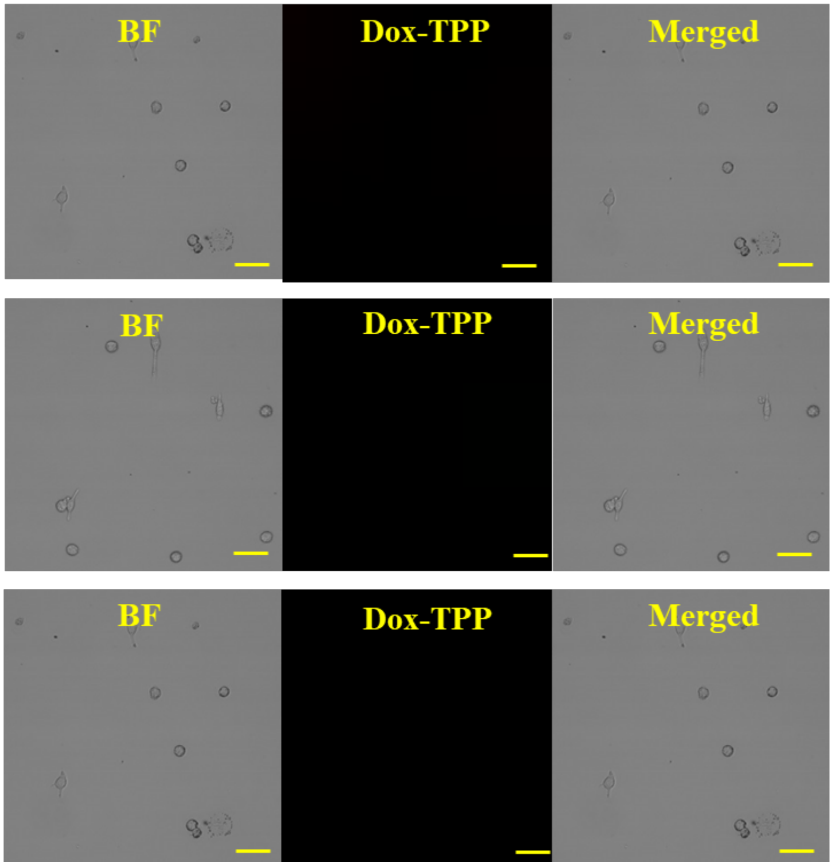

Supplementary Figure 18. Confocal laser scanning microscopy images of differentiated human monocytic leukemia THP-1 cells treated with Dox-TPP loaded crosslinked BSA-PLGA NPs for 1–4 h at pH 7.4. Scale bar = 10 µm.

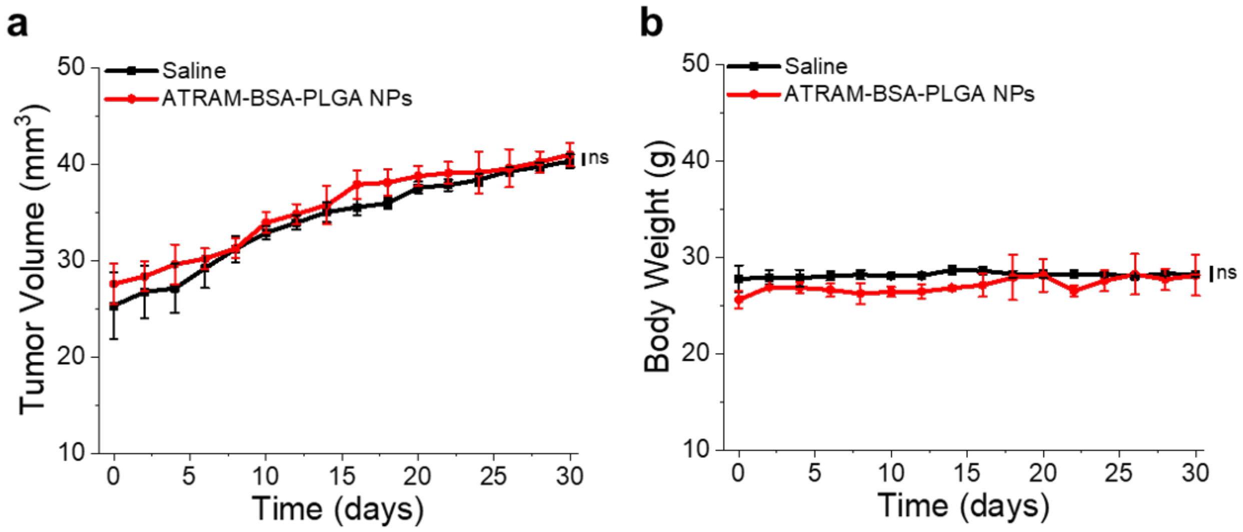

Supplementary Figure 19. Effects of drug-free ATRAM-BSA-PLGA NPs on tumor volume and body weight of 4T1 tumor-bearing mice (n = 4). ns, non-significant (*P* > 0.05).

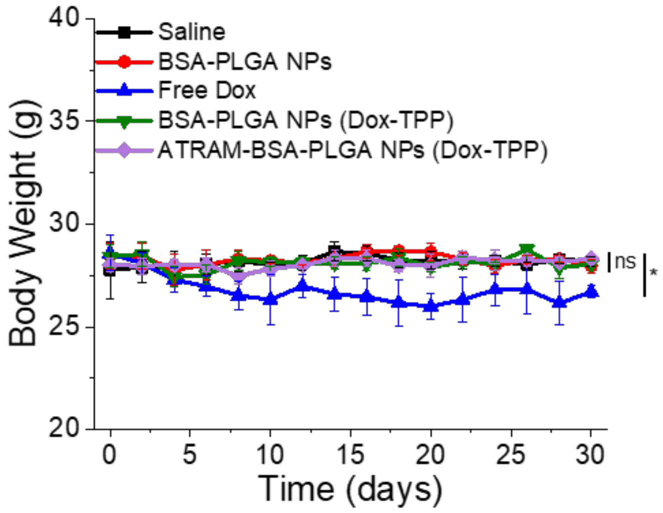

Supplementary Figure 20. Body weight changes of 4T1 tumor-bearing mice following 30 days of treatment with saline, drug-free BSA-PLGA NPs, free Dox or Dox-TPP loaded BSA-PLGA or ATRAM-BSA-PLGA NPs (n = 6). *$P < 0.05$, or non-significant (ns, $P > 0.05$).

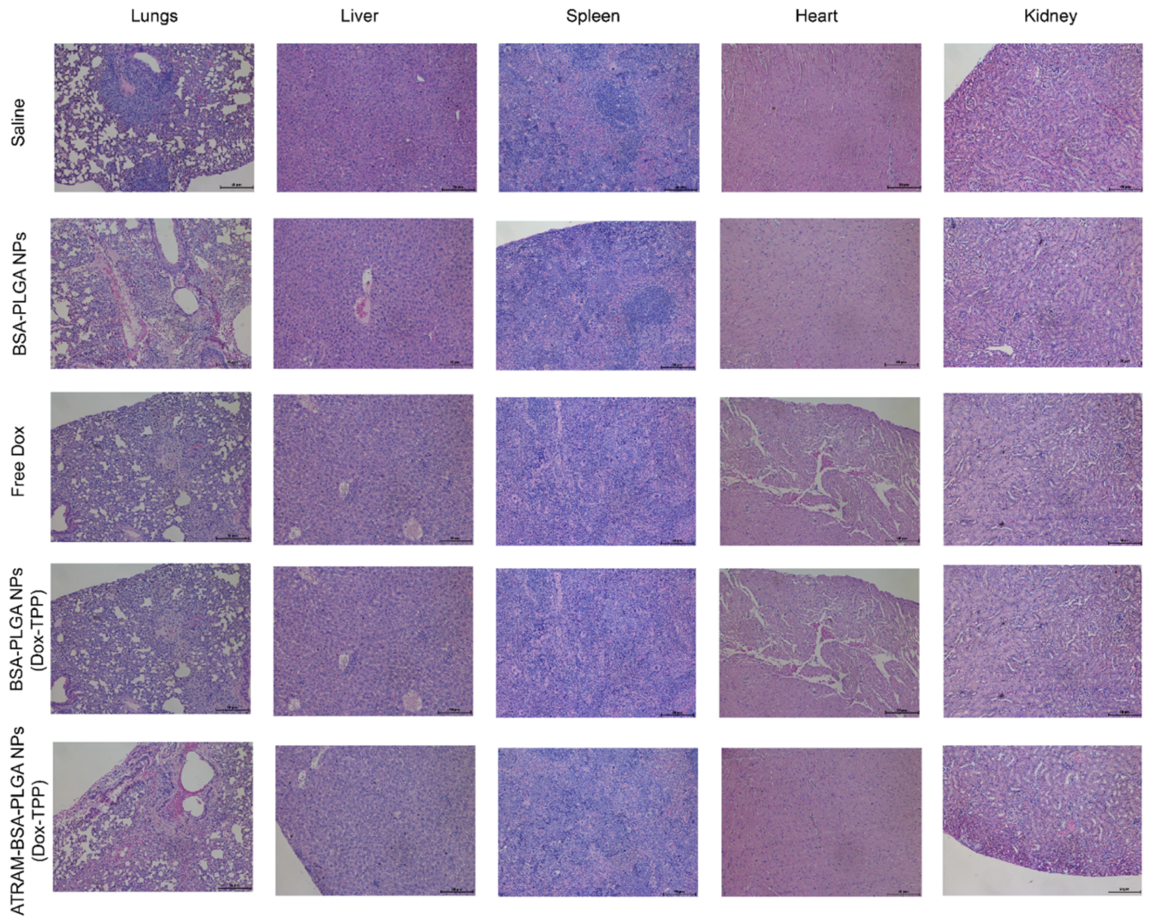

Supplementary Figure 21. Histological analysis of vital organs following treatment with Dox-TPP loaded ATRAM-BSA-PLGA NPs. Hematoxylin and eosin (H&E) staining of lung, liver, spleen, heart and kidney sections from 4T1 tumor-bearing mice after 30 days of treatment with saline, BSA-PLGA NPs, Dox or Dox-TPP loaded BSA-PLGA or ATRAM-BSA-PLGA NPs. Scale bar = 50 μm.

Supplementary Table 1. Drug loading capacity of BSA-PLGA NPs.

Chemotherapeutic Drug	Loading Capacity (wt%)
Doxorubicin-Triphenylphosphonium (Dox-TPP)	1.8
Paclitaxel (PTX)	2.0
Rhodamine B	1.6

The loading capacity of the crosslinked BSA-PLGA NPs was determined using *Equation 2*.

Supplementary Table 2. Hydrodynamic diameters and zeta potentials of PLGA, BSA-PLGA (with and without crosslinking) and ATRAM-BSA-PLGA NPs nanoparticles.

Nanoparticle	Diameter (nm)	Zeta Potential
PLGA	105 ± 2	- 28
BSA-PLGA (without crosslinking)	135 ± 4	- 16
Crosslinked BSA-PLGA	130 ± 4	- 16
ATRAM-BSA-PLGA	131 ± 3	-5 (pH 7.4)<br>+10 (pH 6.5)

Supplementary Table 3. Proteins corresponding to the UniProt Knowledgebase (UniProtKB) accession numbers shown in Figure 2.

UniProtKB Accession Number	Protein
P02769	Serum albumin
P12763	Alpha-2-HS-glycoprotein
Q0IIK2	Serotransferrin
P34955	Alpha-1-antiproteinase
Q9TTE1	Serpin A3-1
P01966	Hemoglobin subunit alpha
P15497	Apolipoprotein A-I
Q3SZR3	Alpha-1-acid glycoprotein
Q58D62	Fetuin-B
Q2KJF1	Alpha-1B-glycoprotein
Q2UVX4	Complement C3
Q5T985	Inter-alpha-trypsin inhibitor heavy chain H2
O94983	Calmodulin-binding transcription activator 2
Q3MHN5	Vitamin D-binding protein
F8WCH0	Actin, gamma-enteric smooth muscle
Q3SZ57	Alpha-fetoprotein
P81644	Apolipoprotein A-II
P02787	Serotransferrin
P02777	Platelet factor 4
Q3T052	Inter-alpha-trypsin inhibitor heavy chain H4
H0YA55	Serum albumin
P17690	Beta-2-glycoprotein 1
P02070	Hemoglobin subunit beta
P01045-1	Kininogen-2
Q03247	Apolipoprotein E
P00735	Prothrombin

Supplementary Note 1

Doxorubicin (Dox), which primarily targets nuclear DNA, is one of the most widely used and potent Food and Drug Administration (FDA)-approved broad-spectrum chemotherapeutic drugs. However, the antitumor property of Dox is often diminished by development of multidrug resistance (MDR) phenotypes in cancer cells.[8,9] Strategies that alter the intracellular distribution of Dox can overcome MDR, thereby increasing the drug's therapeutic efficacy. For instance, conjugating Dox to the mitochondria-specific lipophilic triphenylphosphonium (TPP) cation results in mitochondrial targeting.[1] Upon accumulation in mitochondria, Dox-TPP damages mitochondrial DNA, which lacks the efficient repair mechanisms of nuclear DNA. This, in turn, leads to mitochondrial dysfunction and inhibition of ATP production.[1] Since mitochondrial metabolism and ATP-dependent drug efflux are critical for the survival of drug-resistant cells,[10] mitochondria-targeting enables Dox-TPP to overcome MDR in cancer cells.

Supplementary Note 2

Cancer cells exhibit persistently high levels of reactive oxygen species (ROS) due to the presence of oncogenic mutations that promote aberrant metabolism and protein translation.[11] Since high levels of ROS can damage macromolecules, inactivate enzymes, and trigger senescence and apoptosis, cancer cells counteract the detrimental effects of ROS by producing elevated levels of antioxidant molecules, such as the tripeptide glutathione (GSH; γ-glutamyl-cysteinyl-glycine).[11] Cellular GSH is primarily localized to the cytosol, mitochondria, endoplasmic reticulum and nucleus, and intracellular concentrations of the antioxidant are orders of magnitude higher than those in extracellular fluids.[12] Moreover, studies have reported several fold higher cytosolic levels of GSH in tumors cells compared to healthy cells.[13,14]

A possible explanation for the observed GSH-mediated cargo release from the BSA-PLGA NPs is that the high binding affinity of GSH to BSA leads to formation of stable GSH-BSA complexes[15] on the NP surface, which destabilize the BSA shell. Another possibility is that the crosslinked BSA shell is further stabilized by disulfide bond formation between the exposed Cys34 of adjacent BSA molecules[16] brought into close proximity by crosslinking. GSH would reduce the BSA-BSA disulfide bonds, thereby weakening the BSA shell.

Supplementary Note 3

The highly soluble acidity-triggered rational membrane (ATRAM) peptide interacts with lipid membranes in a pH-dependent manner: at physiological or basic pH, ATRAM binds weakly to the membrane surface in a largely unstructured conformation, whereas under acidic conditions protonation of ATRAM's glutamic acid residues increases the overall hydrophobicity of the peptide and leads to its insertion into lipid bilayers as a transmembrane α-helix.[17,18] The pK_a values of ionizable groups in proteins are highly dependent on the environment: for the glutamic acid side-chain, this value ranges from ~4.0 in solution to > 8.0 in hydrophobic environments.[19,20] Importantly, the measured membrane insertion pK_a of ATRAM is 6.5,[17,21,22] This makes ATRAM ideal for targeting the NPs to cancer cells that reside within the acidic microenvironment of solid tumors.[14,23]

Supplementary Note 4

Tumor-associated macrophages play an important role in cancer survival, proliferation and metastasis.[24] To probe the possible interactions of ATRAM-BSA-PLGA NPs with macrophages within the tumor microenvironment, we attempted to measure the cellular uptake and cytotoxicity of the Dox-TPP loaded NPs in THP-1 cells at pH 6.5. Unfortunately, these experiments were largely unsuccessful due to poor growth of the cells at low pH. Examination of the morphology under the microscope revealed extensive cell damage/death. Moreover, the MTS response of these cells showed significant variability between experiments (and even between different control samples/wells in the same experiment), reflecting the uncontrolled effects of low pH on the cells. Thus, we are unable to comment on the potential interactions between the NPs and tumor-associated macrophages. However, since the vast majority of immune cells (including macrophages) encountered will be in circulation (i.e. at physiological pH),[25,26] the data presented in Figure 7 represents the most relevant condition for assessing the tumor-targeting capabilities of the ATRAM-BSA-PLGA NPs.

Supplementary References

1. Han, M. *et al.* Mitochondrial Delivery of Doxorubicin via Triphenylphosphine Modification for Overcoming Drug Resistance in MDA-MB-435/DOX Cells. *Mol. Pharmaceutics* **11**, 2640–2649 (2014).
2. Silva, C. S. P. C. O., Sousa, F. M. C., Guebitz, G. M. & Paulo, A. C. Chemical modifications on proteins using glutaraldehyde. *Food Technol. Biotechnol.* **42**, 51–56 (2004).
3. Jiang, P., Yu, D., Zhang, W., Mao, Z. & Gao, C. Influence of bovine serum albumin coated poly(lactic-co-glycolic acid) particles on differentiation of mesenchymal stem cells. *RSC Adv.* **5**, 40924–40931 (2015).
4. Wang, Y., Li, P. & Kong, L. Chitosan-Modified PLGA Nanoparticles with Versatile Surface for Improved Drug Delivery. *AAPS PharmSciTech* **14**, 585–592 (2013).
5. Wang, H. *et al.* Enhanced anti-tumor efficacy by co-delivery of doxorubicin and paclitaxel with amphiphilic methoxy PEG-PLGA copolymer nanoparticles. *Biomaterials* **32**, 8281–8290 (2011).
6. Cammarata, C. R., Hughes, M. E. & Ofner, C. M. Carbodiimide Induced Cross-Linking, Ligand Addition, and Degradation in Gelatin. *Mol. Pharmaceutics* **12**, 783–793 (2015).
7. Zorzi, A., Middendorp, S. J., Wilbs, J., Deyle, K. & Heinis, C. Acylated heptapeptide binds albumin with high affinity and application as tag furnishes long-acting peptides. *Nature Communications* **8**, 16092 (2017).
8. Szakács, G., Paterson, J. K., Ludwig, J. A., Booth-Genthe, C. & Gottesman, M. M. Targeting multidrug resistance in cancer. *Nat Rev Drug Discov* **5**, 219–234 (2006).
9. Lage, H. An overview of cancer multidrug resistance: a still unsolved problem. *Cell. Mol. Life Sci.* **65**, 3145 (2008).
10. Dartier, J. *et al.* ATP-dependent activity and mitochondrial localization of drug efflux pumps in doxorubicin-resistant breast cancer cells. *Biochimica et Biophysica Acta (BBA) - General Subjects* **1861**, 1075–1084 (2017).
11. Cairns, R. A., Harris, I. S. & Mak, T. W. Regulation of cancer cell metabolism. *Nature Reviews Cancer* **11**, 85–95 (2011).
12. Montero, D., Tachibana, C., Rahr Winther, J. & Appenzeller-Herzog, C. Intracellular glutathione pools are heterogeneously concentrated. *Redox Biology* **1**, 508–513 (2013).
13. Wang, X. *et al.* Glutathione-Triggered “Off–On” Release of Anticancer Drugs from Dendrimer-Encapsulated Gold Nanoparticles. *J. Am. Chem. Soc.* **135**, 9805–9810 (2013).
14. Palanikumar, L. *et al.* Spatiotemporally and Sequentially-Controlled Drug Release from Polymer Gatekeeper–Hollow Silica Nanoparticles. *Scientific Reports* **7**, 46540 (2017).
15. Jahanban-Esfahlan, A. & Panahi-Azar, V. Interaction of glutathione with bovine serum albumin: Spectroscopy and molecular docking. *Food Chemistry* **202**, 426–431 (2016).

16. Rombouts, I. *et al.* Formation and reshuffling of disulfide bonds in bovine serum albumin demonstrated using tandem mass spectrometry with collision-induced and electron-transfer dissociation. *Scientific Reports* **5**, 12210 (2015).
17. Nguyen, V. P., Alves, D. S., Scott, H. L., Davis, F. L. & Barrera, F. N. A Novel Soluble Peptide with pH-Responsive Membrane Insertion. *Biochemistry* **54**, 6567–6575 (2015).
18. Kalmouni, M., Al-Hosani, S. & Magzoub, M. Cancer targeting peptides. *Cell. Mol. Life Sci.* **76**, 2171–2183 (2019).
19. Harms, M. J. *et al.* The pK(a) values of acidic and basic residues buried at the same internal location in a protein are governed by different factors. *J. Mol. Biol.* **389**, 34–47 (2009).
20. Pace, C. N., Grimsley, G. R. & Scholtz, J. M. Protein ionizable groups: pK values and their contribution to protein stability and solubility. *J. Biol. Chem.* **284**, 13285–13289 (2009).
21. Nguyen, V. P. *et al.* Mechanistic insights into the pH-dependent membrane peptide ATRAM. *Journal of Controlled Release* **298**, 142–153 (2019).
22. Nguyen, V. P., Dixson, A. C. & Barrera, F. N. The Effect of Phosphatidylserine on a pH-Responsive Peptide Is Defined by Its Noninserting End. *Biophysical Journal* **117**, 659–667 (2019).
23. Persi, E. *et al.* Systems analysis of intracellular pH vulnerabilities for cancer therapy. *Nat Commun* **9**, 1–11 (2018).
24. Mantovani, A., Marchesi, F., Malesci, A., Laghi, L. & Allavena, P. Tumour-associated macrophages as treatment targets in oncology. *Nat Rev Clin Oncol* **14**, 399–416 (2017).
25. Rattan, R. *et al.* Nanoparticle-macrophage interactions: A balance between clearance and cell-specific targeting. *Bioorganic & Medicinal Chemistry* **25**, 4487–4496 (2017).
26. Oh, J. Y. *et al.* Cloaking nanoparticles with protein corona shield for targeted drug delivery. *Nat Commun* **9**, 1–9 (2018).
