# Supplementary material for: pH-responsive high stability polymeric nanoparticles for targeted delivery of anticancer therapeutics
Source: Commun Biol. 2020 Mar 3;3:95. doi: 10.1038/s42003-020-0817-4 (PMC7054360; doi:10.1038/s42003-020-0817-4)
Supplement: Supplementary file 2 — Description of Additional Supplementary Files [file 42003_2020_817_MOESM2_ESM.pdf]

Descriptions of Additional Supplementary Files

Data for Figure 1c,e,f,g
Data for Figure 3b,c
Data for Figure 4a,b,c,g
Data for Figure 5g
Data for Figure 6a,b,c,d,e,f,i
Data for Figure 7d,f,g
Data for Figure 8a,c,e,g

Data for Supporting Figure 1b,c
Data for Supporting Figure 3
Data for Supporting Figure 5b,c,d
Data for Supporting Figure 6a,b
Data for Supporting Figure 7b,c,d,e,f,g,h,i,j,k
Data for Supporting Figure 9
Data for Supporting Figure 10
Data for Supporting Figure 15
Data for Supporting Figure 16a,b
Data for Supporting Figure 17
Data for Supporting Figure 19a,b
Data for Supporting Figure 20
