# Supplementary material for: pH-responsive high stability polymeric nanoparticles for targeted delivery of anticancer therapeutics
Source: Commun Biol. 2020 Mar 3;3:95. doi: 10.1038/s42003-020-0817-4 (PMC7054360; doi:10.1038/s42003-020-0817-4)
Supplement: Supplementary file 4 — Reporting Summary [file 42003_2020_817_MOESM4_ESM.pdf]

nature research

Corresponding author(s): Mazin Magzoub

Last updated by author(s): Jan 30, 2020

Reporting Summary

Nature Research wishes to improve the reproducibility of the work that we publish. This form provides structure for consistency and transparency in reporting. For further information on Nature Research policies, see Authors & Referees and the Editorial Policy Checklist.

Statistics

For all statistical analyses, confirm that the following items are present in the figure legend, table legend, main text, or Methods section.

n/a	Confirmed	
☐	☒	The exact sample size (*n*) for each experimental group/condition, given as a discrete number and unit of measurement
☐	☒	A statement on whether measurements were taken from distinct samples or whether the same sample was measured repeatedly
☐	☒	The statistical test(s) used AND whether they are one- or two-sided<br>*Only common tests should be described solely by name; describe more complex techniques in the Methods section.*
☒	☐	A description of all covariates tested
☒	☐	A description of any assumptions or corrections, such as tests of normality and adjustment for multiple comparisons
☐	☒	A full description of the statistical parameters including central tendency (e.g. means) or other basic estimates (e.g. regression coefficient) AND variation (e.g. standard deviation) or associated estimates of uncertainty (e.g. confidence intervals)
☐	☒	For null hypothesis testing, the test statistic (e.g. *F*, *t*, *r*) with confidence intervals, effect sizes, degrees of freedom and *P* value noted<br>*Give P values as exact values whenever suitable.*
☒	☐	For Bayesian analysis, information on the choice of priors and Markov chain Monte Carlo settings
☒	☐	For hierarchical and complex designs, identification of the appropriate level for tests and full reporting of outcomes
☐	☒	Estimates of effect sizes (e.g. Cohen's *d*, Pearson's *r*), indicating how they were calculated

Our web collection on statistics for biologists contains articles on many of the points above.

Software and code

Policy information about availability of computer code

Data collection	For quantitative proteomic analysis, label-free protein quantification was done using the MaxQuant software (version 1.6.5.0) with default parameters. The raw data was searched against the Universal Protein Resource (UniProt) database using the Andromeda search engine.
Data analysis	Statistical analysis was performed using the Prism 7.0 software (GraphPad Software, Inc., La Jolla, CA, USA).

For manuscripts utilizing custom algorithms or software that are central to the research but not yet described in published literature, software must be made available to editors/reviewers. We strongly encourage code deposition in a community repository (e.g. GitHub). See the Nature Research guidelines for submitting code & software for further information.

Data

Policy information about availability of data

All manuscripts must include a data availability statement. This statement should provide the following information, where applicable:

- Accession codes, unique identifiers, or web links for publicly available datasets
- A list of figures that have associated raw data
- A description of any restrictions on data availability

Source data for Figures 1 and 3–8 are available in the Supplementary Data. The proteomics data (Figure 2) has been deposited to ProteomeXchange Consortium via the MassIVE repository (accession code: MSV000084848). All the datasets are also available from the authors on reasonable request.

Field-specific reporting

Please select the one below that is the best fit for your research. If you are not sure, read the appropriate sections before making your selection.

☒ Life sciences ☐ Behavioural & social sciences ☐ Ecological, evolutionary & environmental sciences

For a reference copy of the document with all sections, see nature.com/documents/nr-reporting-summary-flat.pdf

Life sciences study design

All studies must disclose on these points even when the disclosure is negative.

Sample size	Power calculation was used to select sample sizes from the NYU Abu Dhabi Institutional Animal Care and Use Committee (NYUAD-IACUC) Protocol (Protocol No. 18-0001).
Data exclusions	No data was excluded.
Replication	All attempts at replication were successful.
Randomization	For the tumor inhibition studies, tumor-bearing mice were randomized into the treatment groups.
Blinding	For in vitro studies, investigators were blinded for all parts of the experiments (treatment, data acquisition and data analysis), and a different investigator carried out each part.

Reporting for specific materials, systems and methods

We require information from authors about some types of materials, experimental systems and methods used in many studies. Here, indicate whether each material, system or method listed is relevant to your study. If you are not sure if a list item applies to your research, read the appropriate section before selecting a response.

Materials & experimental systems

n/a	Involved in the study	
☒	☐	Antibodies
☐	☒	Eukaryotic cell lines
☒	☐	Palaeontology
☐	☒	Animals and other organisms
☒	☐	Human research participants
☒	☐	Clinical data

Methods

n/a	Involved in the study	
☒	☐	ChIP-seq
☐	☒	Flow cytometry
☒	☐	MRI-based neuroimaging

Eukaryotic cell lines

Policy information about cell lines

Cell line source(s)	All cell lines were from American Type Culture Collection (ATCC).
Authentication	Cell lines were authenticated by Charles River Laboratories.
Mycoplasma contamination	Testing for mycoplasma contamination was done by Charles River Laboratories.
Commonly misidentified lines (See ICLAC register)	N.A.

Animals and other organisms

Policy information about studies involving animals; ARRIVE guidelines recommended for reporting animal research

Laboratory animals	Female C3H/HeJ mice (The Jackson Laboratory, Bar Harbor, ME), 6-8 weeks old.
Wild animals	*Provide details on animals observed in or captured in the field; report species, sex and age where possible. Describe how animals were caught and transported and what happened to captive animals after the study (if killed, explain why and describe method; if released, say where and when) OR state that the study did not involve wild animals.*
Field-collected samples	*For laboratory work with field-collected samples, describe all relevant parameters such as housing, maintenance, temperature, photoperiod and end-of-experiment protocol OR state that the study did not involve samples collected from the field.*

Ethics oversight	All animal experiments were approved by the NYU Abu Dhabi Institutional Animal Care and Use Committee (NYUAD-IACUC; Protocol No. 18-0001), and were carried out in accordance with the Guide for Care and Use of Laboratory Animals.

Note that full information on the approval of the study protocol must also be provided in the manuscript.

Flow Cytometry

Plots

Confirm that:

☒ The axis labels state the marker and fluorochrome used (e.g. CD4-FITC).

☒ The axis scales are clearly visible. Include numbers along axes only for bottom left plot of group (a 'group' is an analysis of identical markers).

☒ All plots are contour plots with outliers or pseudocolor plots.

☒ A numerical value for number of cells or percentage (with statistics) is provided.

Methodology

Sample preparation	Sample preparation details provided in the Experimental Section and were based upon previously published report or manufacturer's protocol.
Instrument	BD FACSAria III cell sorter (BD Biosciences, San Jose, CA).
Software	BD FACSDiva software.
Cell population abundance	Samples were filtered prior to experiments using standard operating procedures. For each experiment, 10,000 cells/sample (3 independent samples) were sorted. Post-sort populations were quantified to determine measured parameters (e.g. cellular uptake or apoptotic fractions).
Gating strategy	10,000 cells/sample (3 biological replicates), gated on live cells by forward/side scatter and propidium iodide (PI) exclusion.

☒ Tick this box to confirm that a figure exemplifying the gating strategy is provided in the Supplementary Information.
